# Peripheral blood mononuclear cells expression of miR-200c, miR-125b, miR-27b, miR-203, and miR-155 in patients with significant or insignificant coronary artery stenosis

**DOI:** 10.1038/s41598-023-45146-8

**Published:** 2023-10-27

**Authors:** Zahra Saadatian, Yaser Mansoori, Lida Nariman-Saleh-Fam, Abdolreza Daraei, Sepideh Zununi Vahed, Shadan Navid, Ziba Nariman-Saleh-Fam

**Affiliations:** 1 Department of Physiology, Faculty of Medicine, Infectious Diseases Research Center, Gonabad University of Medical Sciences, Gonabad, Iran; 2 Noncommunicable Diseases Research Center, Fasa University of Medical Sciences, Fasa, Iran; 3 Faculty of Medicine, Ardabil University of Medical Sciences, Ardabil, Iran; 4 Cellular and Molecular Biology Research Center, Health Research Institute, Babol University of Medical Sciences, Babol, Iran; 5 Kidney Research Center, Tabriz University of Medical Sciences, Tabriz, Iran; 6 Department of Anatomy, Faculty of Medicine, Social Determinants of Health Research Center, Gonabad University of Medical Science, Gonabad, Iran; 7 Women’s Reproductive Health Research Center, Tabriz University of Medical Sciences, Tabriz, Iran; 8 Clinical Research Development Unit, Shohada Hospital, Tabriz University of Medical Sciences, Tabriz, Iran

**Keywords:** Genetics, Cardiology

## Abstract

Coronary artery disease (CAD) is one of the principal causes of death worldwide. Among several predisposing factors, inflammation and inflammatory genes play a significant role in disease pathogenesis. Inflammatory microRNAs, small noncoding RNAs involved in regulating inflammation, are promising candidates for understanding pathogenesis of CAD and developing diagnostic biomarkers. The aim of the study was to evaluate the alteration of miR-200c, miR-125b, miR-27b, miR-203 and, miR-155 in patients suffering from coronary artery stenosis and insignificant coronary artery stenosis compared to healthy subjects. In this study we compared expressions of five inflammatory miRNAs in peripheral blood mononuclear cells (PBMCs) of 72 patients suffering significant coronary artery stenosis (CAD), 74 individuals without coronary artery disease and 30 individuals with insignificant coronary artery stenosis (ICAD). After blood collection, PBMCs were isolated and RNA was extracted. Gene expression levels were assessed by SYBR green based real-time PCR. Statistical analysis was performed using R program. Expression levels of miR-200c, miR-203, and miR-155 were lower in subjects with ICAD than that in CAD patients and subjects of the control group. MiR-125b was downregulated in CAD and ICAD groups compared to the control group. PBMC miR-27b was upregulated in the CAD group as compared to the ICAD and control groups. Receiver operating characteristic curve analysis verified potential of three miRNAs in separating subjects with ICAD from CAD patients and healthy individuals. In conclusion, this original investigation suggested that altered expression of these five miRNAs may serve as a novel diagnostic biomarker discriminating clinical presentations of coronary artery diseases.

## Introduction

Heart diseases such as CAD are known as the most leading cause of death worldwide. CAD is divided into stable angina pectoris (SAP), unstable angina pectoris (UAP) and acute myocardial infarction (AMI) according to clinical manifestation^1^. This disorder is usually initiated with progressive blockage of the heart supplying major blood vessels or coronary arteries, which is called atherosclerosis. Atherosclerosis leads myocardial Perfusion defect and insults chronic inflammation with the recruitment of many inflammatory agents, such as cytokines, chemokines, and PBMCs as well as Lymphocyte and monocyte^2^.

PBMCs may serve as a noninvasive and powerful source for studying human health and disease diagnosis^3^. It has been shown that PBMCs of CAD patients exhibit a profile of inflammatory gene expression^4^. Recently, some reports have indicated the crucial effect of PBMCs microRNA profile in discriminating and early diagnosis of CAD^3^.

MicroRNAs are small non-coding RNAs that can bind to their target genes and attenuate their expression. Their alteration is found in different conditions and might serve as biomarkers^5^. MicroRNAs secreted from monocyte and lymphocytes can fine-tune the expression of inflammatory genes involved in atherogenesis^6^. The expression alteration of microRNAs is conceived in different steps of atherosclerosis and the microRNA signature may be a useful noninvasive tool in early identification of CAD^7^.

NF-κB (nuclear factor kappa B) is the key factor of inflammation activation which induces chemokines and cytokines production^8^. The Toll-like receptor 4 (TLR4)-mediated NF-κB pathway is among signaling pathways implicated in atherosclerotic-related inflammation that has attracted more attention^9^. TLR4 can trigger NF-κB signaling pathway to induce transcription of inflammatory genes^9^. Molecular mechanisms that influence the activity of NF-κB pathway have been shown to play crucial roles in modulating pathological processes in CAD. The current study was aimed at investigating the expression profile of five miRNAs that are involved in regulation of TLR/NF-κB pathways in PBMCs of patients suffering from coronary artery stenosis and insignificant coronary artery stenosis as compared to healthy subjects.

MiR-200c, miR-203, and miR-155 are the regulator of genes involved in inflammation and play roles in the NF-κB pathway. MiR-200c is involved in TLR4 signaling and can reduce NF-κB receptor activity^10^. It also inhibits IL-6, CXCL9, and TNF-α expression in THP-1 cells^10^. MiR-203 can suppress interlukin8 which induces NF-κB activation^11,12^. In addition, it can inactivate the NF-κB pathway through IKKα inhibition^13^. MiR-203 inactivates MEF2C expression that participates in heart development. Also, MEF2c can suppress inflammation through modulating the NF-κB pathway in endothelial cells^14^. MiR-155 is a controversial key regulator of the inflammatory processes and immune responses involved in pro-inflammatory cytokines induction^15^. In endothelial cells, miR-155 activated by TNF-α targets NF-κB, P65 and this negative feedback inhibits atherogenesis by TNF-α suppression^16^. MiR-125b targets TNF-α and TNF receptor-associated factor 6 (TRAF6) in the mouse heart and participates in the NF-kB pathway. Induction of its expression in the mouse heart ceases NF-kB pathway activation through TRAF6 and results in ischemia prevention^17^. MiR-27b belongs to the mir-27 family and is involved in the different biological pathways. In inflammatory processes TLR stimulation drives miR-27b expression in an NF-κB pathway^18^.

In the present study, we aimed to compare the PBMC expression of miR-200c, miR-203, miR-155, miR-125b, and miR-27b in subjects with significant or insignificant stenosis of coronary arteries and healthy subjects.

## Materials and methods

### Study population

As previously explained^19^, the case–control study population consisted of 176 subjects from Shahid Modarres hospital (Tehran, Iran). This included 102 and 74 subjects in the case and control groups, respectively. The case group was comprised of 72 patients suffering significant coronary stenosis [i.e., the CAD group, defined as ≥ 50% stenosis in at-least one coronary artery diagnosed by angiography^20^] and 30 participants with insignificant coronary stenosis [i.e., the ICAD group, < 50% coronary stenosis^20^]. The CAD subgroup was comprised of 32 subjects with unstable angina pectoris (UAP), 20 subjects with stable angina pectoris (SAP) and 20 subjects with acute myocardial infarction (AMI). Patients with a history of any CAD influential disorders as well as heart, liver, renal and infectious disease and neoplasia were excluded from the study. The Gensini score was computed for the evaluation of intensity and location of arteries obstruction. Same hospital clients with normal ECG and stress test sex- and age-matched with case group, randomly selected and compromised our control group and people with previously described exclusion criteria excluded from the control group^19^. Demographic data and clinical characteristics were collected from all groups. Informed consent was acquired from all project attendants. This study was supported by a grant (grant number: 62308) from clinical research development unit, Shohada hospital, Tabriz University of Medical Sciences (Tabriz, Iran) and had been performed in accordance with the Declaration of Helsinki. In addition, this study was approved by the ethics committee of Tabriz University of Medical Sciences (IR.TBZMED.REC.1397.977).

### PBMC isolation and RNA extraction

After collecting ~ 7 ml peripheral blood in EDTA-containing tubes, bloods were diluted with the same volume of the PBS solution. Afterwards, the PBMC isolation was accomplished by adding blood on Ficoll-Paque PLUS (Amersham Pharmacia Biotech, Sweden) and carrying out a centrifuge with 800 g for 20 min. The miRNeasy Mini Kit (Qiagen, Germany) was used for extraction of total RNA including small RNAs^21^. concentration and quality detection of microRNA were carried out by nanodrop. RNA measurement was conducted by measuring ultraviolet absorbance at 260 nm and 280 nm.

### cDNA synthesis and qPCR assays

Each 10 µl reverse transcription reaction mix contained 500 ng RNA. The cDNA synthesis of all miRNAs (Table1) and their reference genes (i.e., RNU48, RNU44, and RNU24) was performed using the standard stem loop RT-qPCR primers and Strand cDNA Synthesis Kit (Clontech, Takara Bio, Japan) according to the its guidelines. the following program was used: 16 °C for 30 min, 42 °C for 30 min, and 5 min at 82 °C. The SYBR green based qPCR reactions were done in duplicates using the Light Cycler 96 instrument (Roche Diagnostics, Mannheim, Germany) for evaluating all gene expressions in 40 cycles at a 95 °C denaturation for 12 min, 60 °C annealing for 30 s and 95 °C extension for 10 s. Each 10 µL qPCR reaction contained 2 µL HOT FIREPol EvaGreen qPCR Mix Plus, 1 µL cDNA, 1 µL of each primer (5 pmol/µL), and 5 µL nuclease‐free water. The melt curve analysis evaluated the specificity of each reaction. The amplification efficiency of each reaction was more than 80%. Cq values and the processed fluorescence information were extracted from Light Cycler 96 software. The R software was applied for analyzing qPCR reactions efficiencies and calculating relative quantities (RQs). ΔCq and normalized relative quantity (NRQ) were computed as explained elsewhere^21,22^.

**Table1:** The sequence of primers used in this study.

miR-155-RT	GTCGTATCCAGTGCAGGGTCCGAGGTATTCGCACTGGATACGACACCCCTA
miR-155-F	CGCCGTTAATGCTAATCGTGAT
miR-155-R	GTGCAGGGTCCGAGGT
miR-125b-RT	GTCGTATCCAGTGCAGGGTCCGAGGTATTCGCACTGGATACGACTCACAA
miR-125b-F	CCGTCCCTGAGACCCTAAC
miR-125b-R	GTGCAGGGTCCGAGGT
miR-27b-RT	GTCGTATCCAGTGCAGGGTCCGAGGTATTCGCACTGGATACGACGCAGAA
miR-27b-F	CGCTTCACAGTGGCTAAGTTCT
miR-27b-R	GTGCAGGGTCCGAGGT
miR-203-RT	GTCGTATCCAGTGCAGGGTCCGAGGTATTCGCACTGGATACGACCTAGTGGTC
miR-203-F	GCCGTGAAATGTTTAGGACCACTA
miR-203-R	GTGCAGGGTCCGAGGT
miR-200c-RT	GTCGTATCCAGTGCAGGGTCCGAGGTATTCGCACTGGATACGACTCCATCAT
miR-200c-F	CGTAATACTGCCGGGTAATGATGG
miR-200c-R	GTGCAGGGTCCGAGGT

### Statistical analysis

R software (version 3.5.1) was applied for data analysis. For testing quantitative variables normality, the Shapiro–Wilk test was used. In order to compare quantitative variable or categorical variable among study groups, Student’s t-test or Mann–Whitney U test and chi-squared tests were used respectively. The Welch’s ANOVA with the Games-Howell post hoc test or One-way ANOVA F test with Tukey’s HSD post hoc test was applied for gene expression comparison between more than two groups whenever expression values were distributed normally. In order to evaluate gene expression comparison between more than two groups without normally-distributed expression values, the Kruskal–Wallis one-way ANOVA with the Conover-Iman test were used. The ROC curve analysis estimated discrimination power of miRNAs and Youden’s method selected suitable threshold.

### Ethics approval

All procedures performed in studies involving human participants were in accordance with the ethical principles and the national standards for conducting medical research in Iran and with the 1964 Helsinki Declaration and its later amendments or comparable ethical standards. The study was approved by Research Ethics Committees of Tabriz University of Medical Sciences (Approval ID: IR.TBZMED.REC.1397.977).

### Informed consent

Informed consent was obtained from all individual participants included in the study.

## Results

The study population was matched in terms of gender, mean age, the proportion of smoking individuals, or the proportion of participants suffering from diabetes, dyslipidemia, obesity, or hypertension (Table 2). Out of 72 patients with significant stenosis, 25 subjects had one vessel disease (i.e., only one vessel with stenosis), 15 subjects had two vessel disease and 32 subjects had three vessel diseases. Furthermore, the CAD group were comprised of 32 UAP, 20 AMI, and 20 SAP patients.

**Table 2:** Demographic and clinical data of the study population.

Parameter	CAD (n = 72)	ICAD (n = 30)	Control (n = 74)	*P*_1_	*P*_2_	*P*_3_
Age (mean ± SD)	60.97 ± 11.99	60.16 ± 7.51	59.87 ± 10.58	0.68	0.56	0.87
Sex (Men/Women)	43/29	19/11	49/25	0.90	0.52	0.95
Hypertension, n (%)	29 (40.2)	10 (33.3)	26 (35.1)	0.66	0.63	1
Dyslipidemia, n (%)	34 (47.2)	10 (33.3)	32 (43.2)	0.28	0.75	0.47
Obese, n (%)	19 (26.3)	7 (23.3)	16 (21.6)	0.94	0.63	1
Diabetes mellitus, n (%)	26 (36.1)	9 (30)	23 (31.0)	0.71	0.63	1
Ever smokers, n (%)	42 (58.33)	19 (63.3)	46 (62.1)	0.80	0.76	1
No. of vessels involved:						
1VSD	25	26	–	–	–	–
2VSD	15	4	–	–	–	–
3VSD	32	0	–	–	–	–
Gensini score	40 (20.2–64)	1 (0.87–1.62)	–	< 0.01	–	–
Collateral circulation (Rentrop grade), n						
0	56 (77.7)	–	–	–	–	–
1	8 (11.1)	–	–	–	–	–
2	8 (11.1)	–	–	–	–	–
Clinical manifestation:						
UAP	32	0	0	–	–	–
AMI (NSTEMI)	20	0	0	–	–	–
SAP	20	0	0	–	–	–
Medications, n (%):						
Antiplatelet therapy	30 (41.6)	6 (20)	7 (9.4)	0.06	< 0.01	0.25
Nitrate	37 (51.3)	5 (16.6)	0	< 0.01	< 0.01	< 0.01
Statin	31 (43)	10 (33.3)	28 (37.8)	0.48	0.63	0.83
B blockers	21 (29.1)	9 (30)	20 (27)	1	0.91	0.94
ACEI or ARB	16 (22.2)	5 (16.6)	15 (20.2)	0.71	0.93	0.88
Calcium antagonist	13 (18)	4 (13.3)	11 (14.8)	0.77	0.76	1

P1: *P*‐value of the test for differences between CAD and ICAD groups. P2: P‐value of the test for differences between CADs and healthy controls. P3: P‐value of the test for differences between ICADs and healthy controls. Abbreviations: ICAD, insignificant coronary artery disease; NSTEMI, non‐ST‐elevation myocardial infarction; NA, not applicable/available; SAP, stable angina pectoris; UAP, unstable angina pectoris.

### MicroRNA concentration and quality detection

The ratio of ultraviolet absorbance at 260 nm/280 nm for all samples was about 2 and they all have suitable purity.

### The PBMC gene expression levels

**Figure 1:**
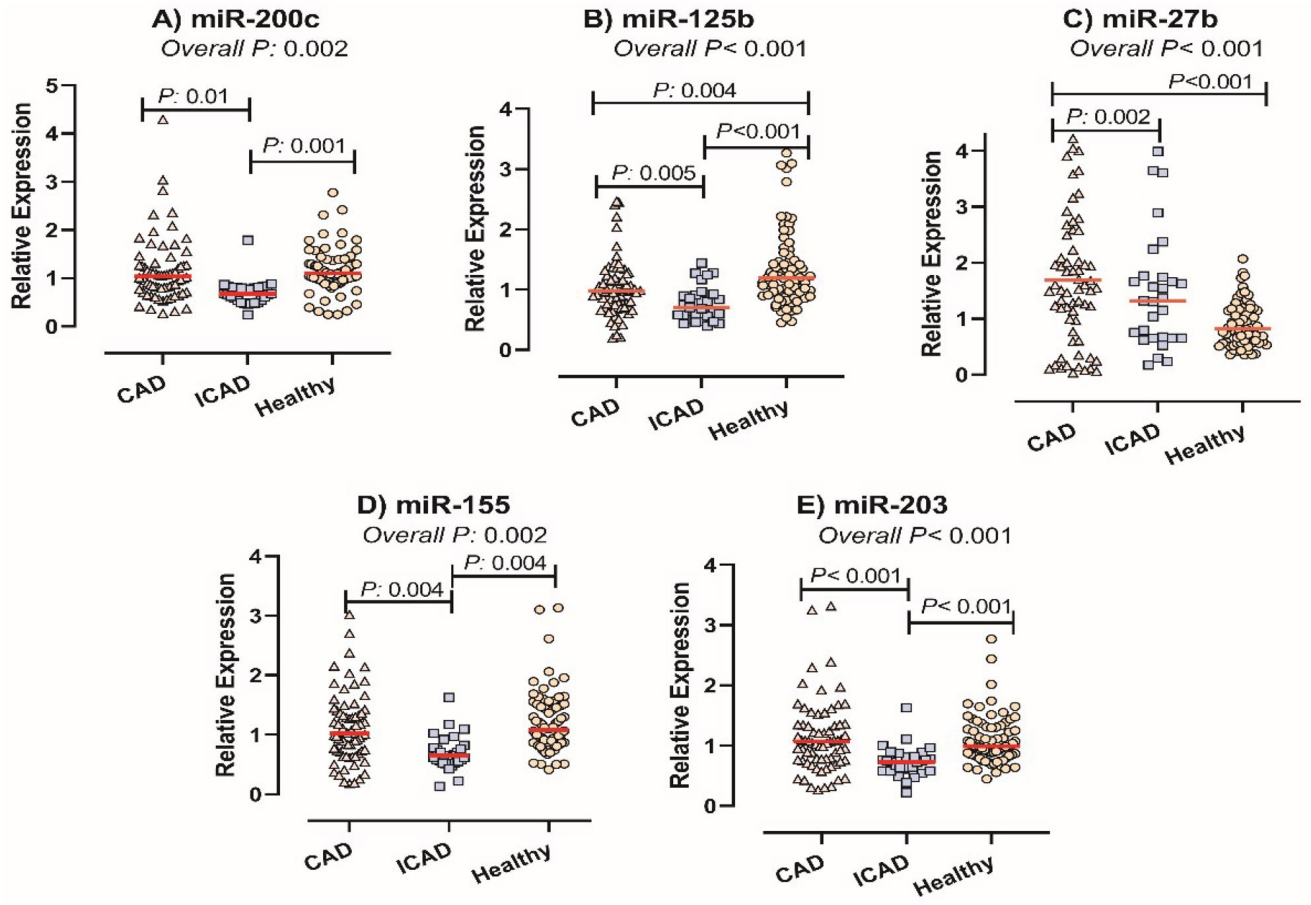
The comparison of the PBMC level of miR-200c (**A**), miR-125b (**B**), miR-27b (**C**), miR-155 (**D**), and miR-203 (**E**) in CAD, insignificant stenosis and the control group. Although no prominent difference was detected between subgroups of the CAD group (i.e., AMI, UAP, and SAP) (all *P* > 0.05), all subtypes had significant increase in terms of the levels of miR-200c, miR-155 and miR-203 compared to subjects of the ICAD group (Fig. 2).

There was no significant difference between subjects of the CAD and the control group in terms of the expression levels of miR-200c, miR-155 and miR-203 (all *P* values > 0.05, Fig. 1). Downregulation of miR-203, miR-200c and, miR-155 was found in insignificant CAD patients as compared to subjects of the CAD and the control group (miR-155: CAD vs. ICAD *P*: 0.004, ICAD vs. controls *P*: 0.004, miR-203: CAD vs. ICAD *P* < 0.001, ICAD vs. controls *P* < 0.001, miR-200c: CAD vs. ICAD *P*: 0.01, ICAD vs. controls *P*: 0.001, Fig. 1). Subjects of the control group had significantly higher expression levels of PBMC miR-125b than subjects of the CAD or ICAD group and CAD patients had higher levels of this miRNA as compared to the ICAD group (Fig. 1, miR-125b: CAD vs. ICAD P: 0.005, CAD vs controls P: 0.004). The PBMC level of miR-27b was lower in the ICAD group and control subjects as compared to the subjects of the CAD group (Fig. 1, miR-27b: CAD vs. ICAD *P*: 0.002, CAD vs. controls *P* < 0.001).

**Figure 2:**
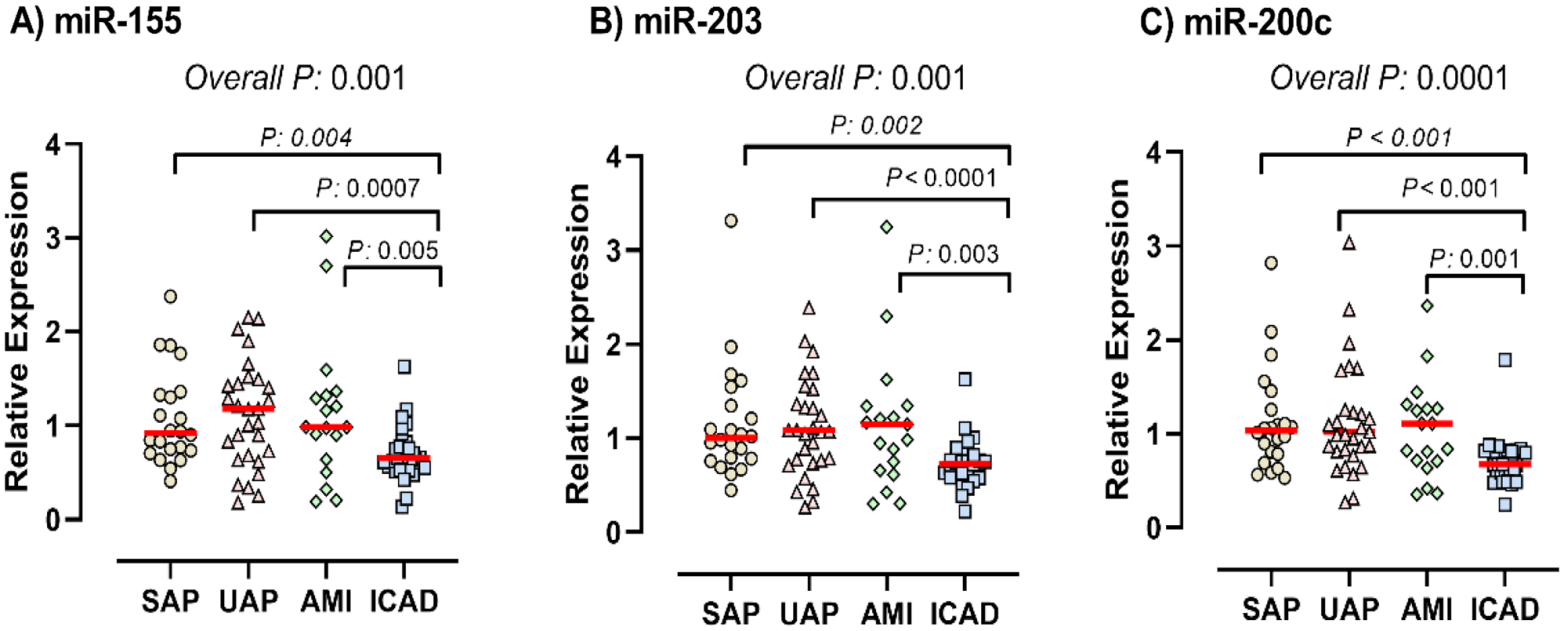
The PBMC level of miR-155 (**A**), miR-203 (**B**), and miR-200 (**C**) in various clinical manifestation of CAD compared with insignificant stenosis.

### Biomarker potential of PBMC expressions

The ROC curve analysis demonstrated that PBMC miR-200c, miR-203 and, miR-155 expression levels may discriminate subjects with insignificant stenosis (i.e., participants of the ICAD group) from patients with significant stenosis with reasonable specificity and sensitivity (miR-203: AUC [95%CI]: 0.75 [0.65 to 0.85], sensitivity: 0.63, specificity: 0.86, miR-155: AUC [95%CI]: 0.74 [0.64 to 0.84], sensitivity: 0.69, specificity: 0.79, miR-200C: AUC [95%CI]: 0.78 [0.69 to 0.87], sensitivity: 0.68, specificity: 0.89). The ROC curve analysis yielded an AUC of 0.68 (95% CI 0.6–0.77) for PBMC levels of miR-27b in discriminating CAD patients from healthy subjects (sensitivity: 0.5, specificity: 0.85). The figure for miR-125b was AUC of 0.64 (95% CI 0.55–0.73, sensitivity: 0.70, specificity: 0.58). PBMC miR-125 and miR-27b levels were also able to discriminate among subjects of CAD and ICAD groups with AUC of 0.69 (miR-125b 95% CI 0.58–0.80, sensitivity: 0.54, specificity; 0.82; miR-27b 95% CI 0.58–0.79, sensitivity: 0.55, specificity: 0.82). Moreover, PBMC levels of miR-200c, miR-203 and miR-155 could distinguish ICAD from AMI, UAP, SAP with acceptable sensitivity and specificity (Table 3).

**Table 3:** Results of the ROC curve analysis showing the potential of PBMC miRNA expression in discriminating various clinical manifestation of CAD from insignificant stenosis.

	miR-155			miR-203			miR-200c		
	AUC (95% CI)	Sensitivity	Specificity	AUC (95% CI)	Sensitivity	Specificity	AUC (95% CI)	Sensitivity	Specificity
SAP vs. ICAD	0.75 (0.62–0.89)	0.63	0.79	0.78 (0.65–0.91)	0.68	0.82	0.80 (0.68–0.93)	0.68	0.96
UAP vs. ICAD	0.75 (0.62–0.88)	0.54	0.96	0.75 (0.62–0.88)	0.61	0.93	0.88 (0.69–0.92)	0.67	0.93
AMI vs ICAD	0.72 (0.54–0.89)	0.79	0.73	0.72 (54–0.89)	0.86	0.63	0.72 (0.55–0.89)	0.96	0.52

SAP: stable angina pectoris; UAP: unstable angina pectoris; AMI: acute myocardial infarction; ICAD: insignificant stenosis; AUC: area under the curve; CI: confidence interval.

## Discussion

Cardiovascular diseases have the most burden of morbidity and mortality worldwide and CAD as a chronic inflammatory disease covers a large part of them^1^. In this investigation, we studied the PBMC expression levels of miR-200c, miR-203, miR-155, miR-125b, miR-27b in CAD, ICAD and healthy subjects. Downregulation of miR-203, miR-200c and, miR-155 was found in insignificant CAD patients while their expression was the same in CAD patients and, healthy controls. Although AMI, UAP and, SAP had higher expression levels than ICAD, there was no significant difference in expression of the three miRNAs between these subtypes. Furthermore, downregulation of miR-125b in ICAD compared with CAD and healthy group was confirmed. Mir-27b showed the highest expression level in CAD and its level were higher in ICAD compared to the controls. The ROC curve analysis suggested that the expression levels of miR-200c, mir-203 and, miR-155 can discriminate ICAD from other groups with acceptable specificity and sensitivity. Furthermore, miR-27b could discriminate CAD patients from controls and ICAD. Also, discriminating among CAD and ICAD groups could be effective with PBMC levels of miR-125b.

Satoh et al. evaluated TLR4-responsive miRNAs such as miR-200 in CAD patients and control groups plasma. Although microarray screening indicated mir-200c downregulation in CAD compared to non-CAD but real-time PCR adoption didn’t show any significant alteration of mir-200c in these groups^23^. miR-200 cluster upregulation suppresses not only inflammatory genes expression including IL-2, IL-4, IL-5, IL-10, IL-13, GM-CSF, INF-γ and, TNF-α but also an angiogenic promoting gene, VEGF-A in MDA-MB231 cells^24^. In another study, on the A549 and HUVEC cell lines, Shi, Liangliang et al. declared miR-200c influences VEGFR-2 expression and elevates the radiosensitivity of cancer cells. Therefore miR-200c inhibition results in angiogenesis increase^25,26^. Kemal Marc Akat et al.^27^ acclaimed miR-203 downregulation in heart failure. However, E.A.Polyakova et al.^28^ noticed miRNA-203 overexpression in serum and cardiomyocytes of patients with multiple coronary artery stenosis compared to controls. Zhi and colleagues have reported the increased levels of miR-203 in the rat heart under hypoxia^29^. Given that, VEGF-A and HIF‐1α are miR-203 targets involved in angiogenesis, miR‐203 hinders angiogenesis and, interferes with neo-angiogenic disorders as well as ischemic heart disease^30,31^. In addition, Liu F and his colleagues informed miR-203 can play role in fine-tuning of placental blood vessels formation by targeting VEGFA and VEGFR2^33^. Julien Faccini et al.^32^ indicated, circulating MiR-155 downregulation is related to CAD. Guo-Fu Zhu et al.^33^ presented miR-155 downturn in PBMC and plasma of CAD patients compared to controls and the low levels of mir-155 are extended in patients with severe stenosis. However, in Leistner et al.^34^ study there are higher levels of mir-155 in trans coronary of patients with increased vulnerable plaques. MiR-155 is involved in HIF1α-mediated angiogenesis. Under the hypoxia condition, MIR-155 is upregulated and drives HIF-1α to fell^45^. VEGF motivates miR-155 expression leading to endothelial cells angiogenesis^35^. Albeit to the above description, some evidences exhibit the antiangiogenic function of mir-155. miR-155 reduces vascular inflammation arrests angiogenesis in endothelial cells of the mouse brain^36,37^. Caballero-Garrido and his colleagues suggested Mir-155 restriction can impede the generation of new vascular and angiogenesis^38^. Angiogenesis impression of miR-155 is specific considering to a cell-type^39^, so it can rationalize our results. Downregulation of miR-125b is found in myocardial tissues of mice with heart failure^40^. Plasma Downregulation of miR-125b compared with noncoronary heart disease is reported in Ding et al.^41^ research. Furthermore, they found a remarkable negative association between Gensini score and the level of miR-125b and miR-125b overexpression is related to Gensini score increase. Chen et al. showed overexpression of miR-125b leads to upregulation of many mitochondria fatty acid metabolism proteins and it is related to cardiac hypertrophy in mice too^42^. Mir-125b could serve as a biomarker in heart defects. This microRNA is a vascular regulator and its increased levels are reported in myocardial ischemia and leads to cardiac self-protection mechanisms activation^43^. After the Heart injury miR-125b level is increased due to myocardial restore^44^. miR-27b is a lipid metabolism regulator and takes apart in atherosclerotic development, inflammation and TGF-β signaling pathway regulation^45,46^. Its elevation was shown in the mice with cardiac hypertrophy and in a heart of a mouse model with a transverse aortic constriction (TAC)-induced cardiac hypertrophy respectively^47,48^. Moreover patients with left ventricular hypertrophy showed high expression of miR-27b in their serum^49^. Furthermore Mir-27b increase was found in patients with adverse paraclinical symptoms of heart failure and it could estimate cardiovascular death^50^.

In conclusion, in this investigation, we reported the variable expression levels of miR-200c, miR-203, miR-155, miR-125b and miR-27b in patients with ICAD. As we described in the introduction, all of these microRNAs participate in NF-kB signaling and are related to inflammation. However, miR-200c, miR-203, miR-155 and miR-125b are involved in angiogenesis. When atherosclerosis is initiated, angiogenesis and novel vessel development are formed to compensate for blood deficiency to the heart muscle. We reported a similar role for mir-196a in ICAD elsewhere^19^. The small plaque of individuals with insignificant coronary stenosis may deteriorate after 18–24 months and cause disabilities^51^. Therefore, the early diagnosis of ICAD is crucial^20,51^. About 30 percent of individuals referred to angiography are diagnosed with ICAD and less than 50 percent of their arteries are blocked^51^. Because of ischemia compensation, angiogenesis and numerous arteries formation is augmented in the early stages of CAD^52^. Accordingly, the expression profile of genes involved in this process is divergent to CAD and healthy controls. It worth mentioning that, to the best of our knowledge, this study is the first inquiry that investigates miR-203, miR-200c, miR-155, miR-125b and miR-27b in PBMCS of ICAD. We hope our findings facilitate the development off noninvasive diagnostic biomarkers. The discrepancy in results of different studies may accentuate the significance of evaluating gene expression profile based on specific cell type and further research is required to unravel the accurate molecular mechanism of these microRNAs, their accompanying components and, details of related pathways in PBMCs of patients with CAD.

## Data Availability

All data generated or analyzed during this study are included in this published article.
